# Effect of Parathyroidectomy on Left Ventricular Mass Index in Patients With Primary Hyperparathyroidism

**DOI:** 10.7759/cureus.33429

**Published:** 2023-01-05

**Authors:** Georgios Tzikos, Ioannis Doundoulakis, Soultana Doutsini, Fotini Adamidou, Stefanos Zafeiropoulos, Leonidas Koliastasis, Christina Manani, Ioannis Pliakos, Theodosios Papavramidis

**Affiliations:** 1 1st Propedeutic Department of Surgery, AHEPA University Hospital of Thessaloniki, Thessaloniki, GRC; 2 1st Department of Cardiology, “Hippokration” Hospital Athens, Athens, GRC; 3 Department of Endocrinology, Diabetes and Metabolism, Hippokration Hospital of Thessaloniki, Greece, Thessaloniki, GRC; 4 Elmezzi Graduate School of Molecular Medicine, Northwell Health, Manhasset, New York, USA, New York, USA

**Keywords:** cohort study, left ventricular ejection fraction, parathyroidectomy, left ventricular mass index, primary hyperparathyroidism

## Abstract

Aim

Primary hyperthyroidism (PHPT) is known to affect left ventricular structure and function and may contribute to increased cardiovascular morbidity and mortality. Whether parathyroidectomy (PTX) reverses left ventricular hypertrophy/remodeling among PHPT patients remains controversial.

Method

In this prospective, single-center study, we enrolled patients with the diagnosis of PHPT who were scheduled for PTX. Patients underwent a complete biochemical workup and an echocardiographic examination at baseline and a six-month follow-up. The primary objective was to compare the left ventricular mass index (LVMI) at baseline and six-month follow-up.

Result

Eighteen patients (15 female, three male, mean age 58.7 years) were enrolled. PTH and serum calcium returned to normal immediately post-PTX and remained normal at six months. LVMI at baseline was within normal limits and reduced further at the six-month follow-up. The left ventricular ejection fraction was in the normal range before the PTX and remained unchanged during follow-up.

Conclusion

Curative PTX reduced LVMI further within the normal range at six months in patients with asymptomatic hyperparathyroidism, providing evidence for benefit in an important non-traditional disease manifestation.

## Introduction

Primary hyperparathyroidism (PHPT) is characterized by inappropriately increased secretion of parathyroid hormone (PTH) from the parathyroid glands. PTH is the key regulator of calcium and phosphate metabolism. The typical electrolyte disorder in PHPT is hypercalcemia, whereas a normocalcemic form of PHPT was recognized almost 15 years ago. The classical clinical presentation is characterized by overt involvement of kidney and bone, while the non-classical disease manifestations include cardiovascular, gastrointestinal, psychiatric, neurocognitive, and quality of life issues [1].

Regarding the cardiovascular manifestations of PHPT, existing data are conflicting; it is argued that patients with PHPT experience cardiovascular morbidity and higher mortality [2-8]. Moreover, the left ventricular structure has been shown to be affected in PHPT and patients develop left ventricular hypertrophy, a strong independent factor of cardiovascular mortality [9-11]. Both increased serum calcium and PTH levels are reported to be associated with increased left ventricular mass [12]. Thus, curative parathyroidectomy (PTX) may lead to left ventricular mass reduction, potentially reversing the unfavorable effects of hypercalcemia and/or PTH on cardiovascular morbidity and mortality. However, observational and randomized trials as well as a meta-analysis have shown controversial results regarding the improvement of cardiovascular outcomes after curative PTX in patients with PHPT [13]. This prospective study aims to compare the left ventricular mass in patients with PHPT before and six months after curative PTX.

## Materials and methods

Design and study population

This is a prospective, single-center, investigator-initiated study, approved by the Ethical Committee of AHEPA University Hospital of Thessaloniki, Greece (29th SesTop/31^st^Con/21.12.2016). The study was also registered in ClinicalTrials.gov prospectively/retrospectively (NCT number: 03091140) and conducted according to the current version of the Declaration of Helsinki. Patients with PHPT due to solitary parathyroid adenoma and without any pre-existing cardiovascular disease were considered eligible. The diagnosis of PHPT was established by the detection of hypercalcemia (normal values 8.2-10.6 mg/dl) along with elevated PTH levels (normal values 1.6-6.9 pmol/l). The patients should meet the following inclusion criteria: i) age more than 18 years old, ii) biochemically proven PHPT, iii) scheduled for a non-emergency parathyroidectomy, and iv) euthyroidism. The patients signed and dated a written informed consent form (ICF) indicating an understanding of the study procedures. Patients were excluded from the study when one or more of the following exclusion criteria was met: i) participation in another clinical trial which may affect this study’s outcomes, ii) recurrent hyperparathyroidism, iii) previous operation at the thyroid or parathyroid glands, iv) history of neck irradiation, v) primary hyperparathyroidism due to hyperplasia or multiple adenomas, vi) secondary hyperparathyroidism, vii) diabetes mellitus, viii) GFR<60ml/min in the last three months, ix) systemic diseases (e.g. infections, neoplasms) and x) abnormal serum albumin.

Initially, a pilot study took place after a small sample of 10 patients had been recruited [14]. After having evaluated the results of the pilot study, the investigators decided to continue the recruitment of new patients in order to improve the strength of the study. All patients were treated with minimally invasive PTX in accordance with the indications for PTX outlined by the Fourth International Workshop [15].

Data collection

Eligible patients were informed about the research protocol by a study investigator. Demographic data and medical records were obtained for each patient. Laboratory results and clinical condition was assessed before PTX, on the first postoperative day, and at the six-month follow-up. Any serious adverse event including the death of any cause, life-threatening events, any hospitalization or prolongation of the existing hospitalization, and events that could result in persistent or significant disability or incapacity, was notified to the Ethics Committee by the investigators. Laboratory testing included complete blood count, serum glucose, creatinine, urea, potassium and sodium, liver biochemistry, lipid panel, serum calcium, albumin and phosphate, 25(OH)2D3, and PTH levels.

Echocardiographic evaluation was conducted at the hospital’s echocardiography core lab according to the guidelines of the American Society of Echocardiography using a GE Vivid S5 ultrasound machine. Left ventricular end-diastolic dimension (LVEDD), interventricular septal thickness at end-diastole (IVSd), and posterior wall thickness at end-diastole (PWd) were measured. Left ventricular mass (LVM) was calculated using the following formula: LVM (g) = 0.8 x 1.04 [(LVEDD + IVSd + PWd)^3^ - (LVEDD)^3^] + 0.6. The left ventricular mass index (LVMI) was calculated as LVM divided by body surface area [16, 17]. The normal reference range was 43-95g/m^2^ for women and 49-115g/m^2^ for men. Left Ventricular Ejection Fraction (LVEF) was measured based on Simpson’s biplane formula and it was considered normal when measured ≥55% [17].

Statistical analysis

After power analysis, it was estimated that a sample size of 18 patients would provide a power of 80% with a level of significance of 0.05 (two sided), for detecting an effect size of 0.75 between paired values. A drop-out rate of 10% was assumed and, thus, a total of 20 patients were needed.

IBM SPSS Statistics software (25^th^ edition) was used for the statistical analysis. Statistical significance was set to p<0.05. Shapiro-Wilk test was used to assess the normality of data distribution. The results are presented as means ± standard deviation, when normality was assumed. Student-t test for paired data was used to compare the means of continuous variables in two different times, given that normality was assumed.

## Results

A total of 20 patients were enrolled in the study and underwent curative PTX. Two patients were excluded from the analysis: one patient was found to have an atypical adenoma on histology and another patient was lost to follow-up. Eighteen patients (15 female, mean age 58.7 years) were included in the analysis. Their baseline characteristics are presented in Table 1. Three patients had a history of primary hypertension and were on treatment with an angiotensin II receptor blocker (Olmesartan), while the remaining 15 patients were normotensive.

**Table 1:** Baseline characteristics of patients who completed follow-up. BMI: Body Mass Index, SD: Standard Deviation

Characteristics	Patients (N=18)
Female, (%)	15 (83.3)
Age (years) [SD]	58.7 [± 10.1]
Height (m) [SD]	1.59 [± 0.05]
Weight (kg) [SD]	65.8 [± 11.9]
BMI (kg/m^2^) [SD]	26.3 [±4.9]
Nationality	18/18 Greek
PTH (pmol/lit) [SD]	11.87 [±3.08]
Serum calcium (mg/dl) [SD]	11.24 [±0.46]

After PTX, mean PTH and albumin-corrected serum calcium levels normalized, immediately postoperatively and at six months. LVMI was normal prior to PTX in all 18 subjects. However, it was reduced significantly six months after curative PTX. LVEF was normal at the initial assessment and remained normal six months after PTX (p=0.22) (Table 2). The patients receiving antihypertensive therapy continued taking their medication unchanged.

**Table 2:** PTH, serum calcium levels, LVMI and LVEF before and 6 months after PTX. PTX: parathyroidectomy, SD: Standard Deviation, LVMI: Left Ventricular Mass Index, LVEF, Left Ventricular Ejection Fraction, N/M: not measured

Patients (Ν=18)	Before PTX	Immediately post-PTX	6 months after PTX	P-value
	(N=18)	(N=18)	(N=18)	
PTH (pmol/lit) [SD]	11.87 [±3.08]	2.92 [1.57]	5.31 [0.89]	<0.001
Serum Calcium (mg/dl) [SD]	11.24 [±0.46]	9.49 [0.85]	9.22 [0.57]	<0.001
LVMI (g/m^2^) [SD]	68.20 [±16.87]	N/M	62.90 [14.16]	0.04
LVEF (%) [SD]	67.9 [±3.8]	N/M	68.6 [±4.3]	0.22

## Discussion

In this single-center observational study, curative PTX was associated with a significant reduction of LVMI within the normal limits. LVEF was normal and did not change significantly after surgery.

Long-term observations confirm that untreated hyperparathyroidism represents an adverse prognostic risk factor, associated with premature cardiovascular death [16]. However, it is not clear whether the calcium and phosphate imbalance and/or elevated PTH per se are responsible for cardiac remodeling. The role of Ca^2+^ channels in the pathogenesis of cardiac hypertrophy has been extensively investigated but no safe conclusions can be extracted [17, 18]. Experimental models surmise that calcium release triggered by excess PTH results in increased protein kinase C activity, leading to myocardial hypertrophy [19, 20]. Cardiac hypertrophy seems to be the phenotypic reflection of multiple pathophysiologic alterations. Importantly, the correlation between hypercalcemia, hyperparathyroidism, and arterial hypertension is well-established, and left ventricular hypertrophy is the physical adaptation of cardiac myocytes to hypertension [21-23]. Thus, the effect of PTX on LVM may be mediated by an improvement in blood pressure following PTX.

Available data regarding structural heart involvement in PHPT patients remain controversial. However, cardiac dysfunction has been widely reported among the published studies. In a recent study, left ventricle systolic and diastolic functions were assessed with specific echocardiographic techniques, namely the tissue Doppler imaging (TDI) and strain and strain rate echocardiography to evaluate myocardial systolic and diastolic function in 31 PHPT patients and 29 healthy controls and revealed that PHPT patients without any known underlying cardiovascular disease experience both subclinical systolic and diastolic heart dysfunction [24]. However, another study published previously, also using TDI for cardiac function evaluation, reported that PHPT patients without cardiovascular risk factors had normal global systolic and diastolic function and cardiac morphology [25]. Data from the case-control study by Walker et al, indicated that mean LVMI was found within the normal range in patients with PHPT and did not differ compared to healthy controls (98 ± 23 vs. 96 ± 24 g/m^2^, p=0.69) [26]. Persson et al. evaluated the changes of echocardiographic variables reflecting the effect of PTX on myocardial function in patients with mild PHPT compared to patients with PHPT randomized to observation. They found minor differences between the two groups, including a borderline significant postoperative amelioration of LVMI at two years compared to active surveillance (p=0.066), while diastolic dimensions of the interventricular septum reduced significantly (p<0.01) only in the patients undergone surgery [27].

Many published studies support that left ventricular hypertrophy prevalence among patients with hyperparathyroidism is high and possibly reversible after PTX, at least in the short term [28]. In addition, the meta-analysis of McMahon et al, including four randomized control trials and 11 observational studies, reported that LVM reduced by 11.6g/m^2^ (12.5%) on average after curative PTX in the immediate postoperative period but this effect was not sustained beyond six months [13]. This finding implies a subtle cardiovascular dysfunction in PHPT that is ameliorated early after PTX, during the cardiovascular adaptation to diminished calcium and PTH levels [29, 30]. In our study, LVMI reduction and a stable LVEF were confirmed.

This study is subject to the limitations associated with real-world observational research. The main limitation is the small subject sample that does not allow safe conclusions. Duration of follow-up was limited to six months after surgery and it is questionable whether the benefit of PTX on LVMI wanes over time. Except for IVSd, PWd, LVEDD, LVMI, and LVEF no other echocardiographic analysis was made and no valve assessment was conducted. LVEF should ideally be combined with parameters such as longitudinal strain for a more thorough study of the systolic function. In addition, only patients with mild types of PHPT were included. However, nowadays, we rarely assess patients presenting with the classical form of PHPT. Furthermore, differences in outcome between men and women could not be made, since the majority of subjects were women. Finally, although adjustments for blood pressure measurements and antihypertensive medications were not made, the small proportion of patients with hypertension did not influence the results.

## Conclusions

In conclusion, curative PTX reduced mean LVMI at six months, in a small sample of patients with mild PHPT. LVEF remained in the normal range. These results are in accordance with previous findings suggesting a favorable, short-term effect of PTX on left ventricular function, but should be interpreted with caution and larger prospective studies in patients with mild, asymptomatic hyperparathyroidism are necessary for safe conclusions. However, this study primarily tried to raise the issue that cardiovascular impairment should be seriously under consideration when dealing with patients with PHPT.
